# Probiotics and Risk of Death, Necrotizing Enterocolitis, and Sepsis in Very Preterm Infants

**DOI:** 10.1001/jamanetworkopen.2026.35560

**Published:** 2026-09-23

**Authors:** Ayoub Mitha, Sofia Söderquist Kruth, Emma Sinervo, Magnus Domellöf, Björn Brindefalk, Thomas Abrahamsson, Stefan Johansson, Alexander Rakow

**Affiliations:** 1 Division of Clinical Epidemiology, Department of Medicine Solna, Karolinska Institutet, Stockholm, Sweden; 2 Department of Neonatal Medicine, Bretonneau Hospital, François Rabelais University, CHU Tours, Tours, France; 3 Université Paris Cité and Université Sorbonne Paris Nord, Inserm, INRAE, Center for Research in Epidemiology and Statistics, Obstetric, Perinatal, Paediatric Life Course Epidemiology Research Team, Paris, France; 4 Department of Women’s and Children’s Health, Karolinska Institutet, Stockholm, Sweden; 5 Women’s Health and Allied Health Professional Theme, Karolinska University Hospital, Solna, Stockholm, Sweden; 6 Centre for Epidemiology and Community Medicine, Region Stockholm, Stockholm, Sweden; 7 Deptartment of Clinical Sciences, Pediatrics, Umeå University, Umeå, Sweden; 8 Department of Molecular Biosciences, The Wenner-Gren Institute, Stockholm University, Stockholm, Sweden; 9 Department of Ecology, Environment and Geoscience, Umeå Universitet, Umeå, Sweden; 10 Department of Biomedical and Clinical Sciences, Linköping University and Crown Princess Victoria Children’s Hospital, Linköping, Sweden; 11 Department of Neonatology, Karolinska University Hospital, Stockholm, Sweden

## Abstract

**Importance:**

Probiotics are reported to benefit preterm infants, but population-based clinical evidence is limited. In 2020, the Swedish Neonatal Society issued a national recommendation to supplement very preterm infants (28 weeks 0 days’ to 31 weeks 6 days’ gestation) with probiotics.

**Objective:**

To investigate whether probiotic supplementation was associated with risk of death, necrotizing enterocolitis (NEC), and/or culture-proven sepsis in infants born very preterm.

**Design, Setting, and Participants:**

This population-based cohort study examined data from the Swedish Neonatal Quality Register from January 1, 2017, through December 31, 2024. Live-born, very preterm infants, excluding those who died within the first 3 days of life or due to congenital or chromosomal abnormalities, were included.

**Exposure:**

Daily probiotic supplementation of 1 billion colony-forming units of freeze-dried *Bifidobacterium infantis*, *Bifidobacterium lactis*, and* Streptococcus thermophilus* initiated following birth and discontinued at postmenstrual week 34.

**Main Outcomes and Measures:**

Composites of death and/or NEC (Bell stage 2-3), death and/or surgical NEC (Bell stage 3), and death and/or culture-proven sepsis were assessed. Modified Poisson regression, propensity score–matched, and inverse probability of treatment weighting analyses were used for replication analyses. A multivariate bayesian modeling analysis accounting for the association between NEC and mortality was performed to estimate the association between probiotic exposure and mortality.

**Results:**

Among 4695 infants (mean [SD] gestational age, 30.2 [1.1] weeks; 2550 male [54.3%]), probiotic exposure increased from 0% before the national recommendation in 2020 to 16.9% in 2020 and a mean (SD) of 64.8% (4.7%) in 2021 to 2024. Probiotic supplementation vs no supplementation was associated with lower risk of death and/or NEC (rate, 0.8% vs 3.4%; adjusted relative risk [ARR], 0.28 [95% CI, 0.14-0.56]). When assessed separately, risks of death (rate, 0.3% vs 1.4%; ARR, 0.16 [95% CI, 0.06-0.45]) and NEC (rate, 0.6% vs 2.5%; ARR, 0.25 [95% CI, 0.10-0.61]) were both lower in the probiotic group. Multivariate bayesian modeling suggested that a substantial proportion of the observed mortality reduction was mediated via reduced NEC incidence (42.3% [credible interval, 16.1%-100%]). Probiotic supplementation was not associated with death and/or culture-proven sepsis (rate, 3.4% vs 4.4%; ARR, 0.86 [95% CI, 0.58-1.26]).

**Conclusions and Relevance:**

This cohort study of very preterm infants found that implementation of a multistrain probiotic was associated with a significant reduction in death and/or NEC, which may help inform shared decision-making between clinicians and families. These results should not be generalized to extremely preterm infants (<28 weeks’ gestation).

## Key Points

**Question:**

Is there an association of multistrain probiotic supplementation in very preterm infants (28 weeks 0 days’ to 31 weeks 6 days’ gestation) with risk of death, necrotizing enterocolitis (NEC), and/or sepsis?

**Findings:**

In this cohort study of 4695 very preterm infants born between 2017 and 2024, probiotic supplementation was associated with reduced risks of death and/or NEC (0.8% vs 3.4%). The risk of death and/or sepsis remained unchanged (3.4% vs 4.4%).

**Meaning:**

These findings suggest that probiotic supplementation may improve outcomes in very preterm infants, but caution is warranted when extrapolating these findings to other populations, including extremely preterm infants (<28 weeks’ gestation).

## Teaser

This cohort study examines the association of probiotic supplementation with risk of death, necrotizing enterocolitis (NEC), and/or culture-proven sepsis in infants born very preterm in Sweden.

## Introduction

Prevention of necrotizing enterocolitis (NEC) remains challenging.^1,2^ In addition to breast milk feeding and potentially decreased antibiotic use, supplementation of probiotic bacteria has emerged as a strategy to reduce NEC incidence.^3,4,5^ Probiotics have been linked to numerous gastrointestinal benefits in animal models, including reduced intestinal permeability, stimulation of immune functions, and inhibition of pathogenic bacteria.^6^ Given the central role of intestinal immaturity and dysbiosis in the pathogenesis of NEC,^7^ these mechanisms provide a biological rationale for probiotic supplementation of preterm infants with immature gut structure and function.

Over the past decade, a growing body of evidence has suggested that specific probiotic strains better reduce the incidence of NEC and all-cause mortality in preterm infants.^3,4,8,9,10^ However, results have varied across studies due to heterogeneity in strain selection, dosing regimens, timing of initiation, duration of supplementation, and study populations.^3^ In addition, concerns regarding product quality and safety have contributed to wariness in implementing probiotics in clinical practice.^11,12,13^ Consequently, substantial variability in probiotic use exists across neonatal units worldwide, including differences in whether probiotics are used at all, which preparations are selected, and which infants are targeted.^14,15^

In response to the debate about the evolving evidence base, the European Society for Paediatric Gastroenterology, Hepatology and Nutrition issued a position statement in 2020 conditionally supporting use of specific probiotic strains in preterm infants.^16^ In line with these European recommendations, the Swedish Neonatal Society issued a national recommendation in 2020 to provide probiotics to very preterm infants born between 28 weeks 0 days’ and 31 weeks 6 days’ gestation.^17^ Given the insufficient evidence for extremely preterm infants (<28 weeks’ gestation) at that time, this patient group was not included in the recommendation but, instead, included in a national randomized clinical trial (Probiotics in Extreme Prematurity in Scandinavia).^18^ Implementation after this nationwide recommendation provides an opportunity to evaluate associations between probiotic supplementation and death, NEC, and sepsis in a population-based setting in which exposure and outcome data were prospectively recorded in a national register.

## Methods

### Study Design and Population

This cohort study used data from the Swedish Neonatal Quality Register (SNQ), capturing all admissions to the 38 neonatal intensive care units (NICUs) in Sweden and prospectively recording data at time of discharge or through continuous recording during the length of stay.^19^ The study received ethical approval from the Swedish Ethical Review Authority (Dnrs 2024-06745-02 and 2025-04906-02). Informed consent was waived by the Swedish Ethical Review Authority as all parents received information regarding the recording of data to the SNQ with a right to opt out and have all personal information removed. This study followed the Strengthening the Reporting of Observational Studies in Epidemiology (STROBE) reporting guideline for cohort studies.

Maternal, pregnancy, and delivery data were automatically extracted from medical records and transferred via the Swedish Pregnancy Register^20^ to SNQ. The SNQ and Swedish Pregnancy Register use the unique Swedish personal identification number for both mother and infant for linkage.^21^ Records of very preterm infants were extracted from the SNQ of infants live born in Sweden after 28 weeks 0 days’ to 31 weeks 6 days’ gestation from January 1, 2017, through December 31, 2024. The study population excluded infants who died within the first 3 days of life, died at any time point due to congenital or chromosomal abnormalities, or had missing data on confounders in the regression model (birth weight, Apgar score). Race and ethnicity are not recorded in SNQ and, therefore, such information was not available in our dataset.

### Enteral Nutrition Practice

During the study period, early feeding of very preterm infants was predominantly based on the mother’s own or donor breast milk. Formula use was rare. Enteral feeding was initiated during the first hours after delivery and gradually increased depending on feeding tolerance. Fortification of enteral feeds were tailored individually based on breast milk analyses (weekly or every 2 weeks), with the help of computer software (Nutrium; Trients AB).

### Exposure

Prior to 2020, probiotics were not used in Swedish NICUs. In 2020, probiotic supplementation was introduced in Swedish NICUs as standard of care for very preterm infants according to a national guideline issued by the Swedish Neonatal Society^17^ based on recommendations by the European Society for Paediatric Gastroenterology, Hepatology and Nutrition.^16^ A freeze-dried blend of *Bifidobacterium infantis *Bb-02 (DSM 33361), *Bifidobacterium lactis* (BB-12), and *Streptococcus thermophilus* (TH-4) totaling 1 billion colony-forming units in maltodextrin powder was used (ProPrems; Neobiomics AB), diluted in 2 to 3 mL of breast milk, and given once daily. Per the national guideline, probiotics were to be initiated before the fourth day of life and discontinued at postmenstrual week 34. We categorized probiotic supplementation depending on whether each infant was supplemented with probiotics based on a tick box recording in SNQ.

### Outcomes

The primary outcomes were composites of death and/or NEC and death and/or culture-proven late-onset sepsis, as death was regarded as a competing risk. The subcomponents were also assessed separately. Data on deaths during the NICU stay, including cause and age, were recorded in SNQ and validated against the Swedish Death Registry.^22^ Mortality analyses included only deaths occurring after the first 3 days of life and not attributable to congenital or chromosomal abnormalities, as such deaths were regarded as unrelated to probiotics supplementation.

Necrotizing enterocolitis was defined as Bell stage 2 or 3, as recorded in SNQ as any NEC (Bell stage 2-3) or surgical NEC (Bell stage 3) according to the NEC definition in the SNQ handbook. Bell stage 2 or 3 NEC was diagnosed clinically or radiologically, at surgery, or at autopsy. The criteria for a diagnosis were symptoms of bile-stained ventricular aspirate; abdominal distension, discoloration, or pain; or visible or occult blood in feces together with radiological findings of pneumatosis intestinalis or air in the portal vein and/or pneumoperitoneum. Per the SNQ handbook, the following were not defined as NEC: Bell stage 1 and infants with an initial NEC diagnosis in which surgery or autopsy later showed focal gastrointestinal perforation or gastrointestinal malformation. To address potential misclassification,^23^ NEC in the composite outcome of death and/or NEC was analyzed both as any NEC (Bell stage 2-3) and as surgical NEC (Bell stage 3) based on the SNQ data.

Late-onset sepsis was considered as registration of at least 1 culture-proven sepsis event after 3 days of life, defined as follows in the SNQ handbook: clinical symptoms, 1 or more positive blood cultures deemed significant, laboratory results compatible with sepsis, intravenous antibiotic therapy of at least 5 days, and no NEC diagnosis. Our dataset had no data about causative bacteria.

### Statistical Analysis

Given the low frequency of missing data, the primary analyses used complete case data, excluding infants with unrecorded birth weight and unrecorded Apgar score. Infants exposed or not exposed to probiotics were compared with respect to perinatal and neonatal characteristics. Characteristics considered to be associated with both probiotic exposure and outcomes were regarded as potential confounders (eFigure 1 in Supplement 1). To assess the association between probiotic exposure and the composite outcomes and their subcomponents, we used modified Poisson regression with robust standard errors. Analyses were adjusted for gestational age (days), birth weight *z* score,^24^ 5-minute Apgar score, surfactant treatment (yes or no), and year of birth (continuous variable), and hospital clustering was accounted for using cluster-robust standard errors.

We performed additional analyses to assess robustness of the findings. To control in a different way for confounding, we performed propensity score (PS)–matched analyses in a subcohort of infants using 1:1 nearest-neighbor matching without replacement, a logit PS, and a caliper width of 0.1. Matching variables included the covariates in the regression model; PS-matched analyses were controlled for clustering by hospital; and balance was evaluated using standardized mean differences before and after matching. Additionally, an inverse probability of treatment weighting analysis was performed to ensure a balanced risk distribution between the probiotics and no probiotics groups, with stabilized weights, truncated at the 1st and 99th percentiles, derived from the same PS model. To more directly model the joint structure of adverse outcomes, NEC, death, and sepsis were modeled using a bayesian multivariate regression (eAppendix in Supplement 1) with correlated residuals (brms, version 2.21),^25^ including probiotic exposure, surfactant, gestational age, birth weight *z* score, and Apgar score, as estimators and hospital and birth year as random intercepts. Penalized spline terms were used for continuous variables. The model was fit using Hamiltonian Monte Carlo via the cmdstanr interface (4 chains × 4000 iterations), with weakly informative priors and adapt_delta = 0.95. Residual correlations between outcomes were estimated directly.

Analyses were performed using R, version 4.5.1 (R Foundation for Statistical Computing) packages sandwich, version 3.1-1^26^; clubSandwich, version 0.6.1^27^; and MatchIt, version 4.7.2.^28^ A 2-sided *P* < .05 was considered statistically significant.

## Results

### Patients

The SNQ included 4922 very preterm infants live born in Sweden after 28 weeks 0 days’ to 31 weeks 6 days’ gestation between 2017 and 2024. Excluded infants included 63 who died within the first 3 days of life, 11 who died of congenital or chromosomal abnormalities, 49 with missing birth weight, and 104 with missing Apgar scores, resulting in an analytic cohort of 4695 infants (mean [SD] gestational age, 30.2 (1.1) weeks; 2145 female [45.7%] and 2550 male [54.3%]) (Table 1). The probiotics group included 1571 infants (33.5%), and the no probiotics group included 3124 infants (66.5%). Infants supplemented with probiotics compared with those not supplemented were more often born in university hospitals (711 [45.3%] vs 1271 [40.7%]), had a lower gestational age (mean [SD], 30.2 [1.1] vs 30.3 [1.1] weeks), and had a lower Fenton birth weight *z* score (mean [SD], −0.33 [1.29] vs −0.20 [1.45]). Infants supplemented with probiotics compared with those not supplemented were less likely to have an Apgar score of less than 7 at 5 minutes (213 [13.6%] vs 590 [18.9%]), had a shorter treatment time with invasive ventilation (mean [SD], 3.5 [4.8] vs 5.9 [15.2] days), and had a longer treatment time with noninvasive ventilation (mean [SD], 16.7 [16.6] vs 13.8 [16.5] days). A total of 49 infants (1.0%) died in the study population at a median age of 11 days (range, 3-145 days), 18 of whom (36.7%) had NEC recorded as cause of death (eTable 1 in Supplement 1). In a subcohort of 1908 infants (40.6%) born in 2020 to 2024, baseline characteristics were similar compared with the full study population (eTable 2 in Supplement 1).

**Table 1.** Characteristics of Very Preterm Infants Receiving or Not Receiving Probiotic Supplementation, Sweden 2017-2024 (N = 4695)

Characteristic	Infants, No. (%)		SMD^a^
	No probiotics (n = 3124)	Probiotics (n = 1571)	
Gestational age, mean (SD), wk	30.3 (1.1)	30.2 (1.1)	0.10
Birth weight, mean (SD), Fenton *z* score	−0.20 (1.45)	−0.33 (1.29)	0.09
Birth weight for gestational age			
Appropriate	2611 (83.6)	1345 (85.6)	0.06
Large	118 (3.8)	31 (2.0)	0.11
Small	395 (12.6)	195 (12.4)	0.02
Infant sex			
Female	1438 (46.0)	707 (45.0)	0.02
Male	1686 (54.0)	864 (55.0)	0.02
Singleton	2327 (74.5)	1194 (76.0)	0.04
Vaginal delivery	906 (29.1)	472 (30.1)	0.02
Birth hospital			
Level 2	1853 (59.3)	860 (54.7)	0.09
Level 3	1271 (40.7)	711 (45.3)	0.09
Apgar score <7 at 5 min	590 (18.9)	213 (13.6)	0.14
Apgar score <4 at 5 min	84 (2.7)	47 (3.0)	0.02
Transient tachypnea^b^	1265 (40.5)	693 (44.1)	0.07
Respiratory distress syndrome^c^	1580 (50.6)	777 (49.4)	0.03
Surfactant treatment	907 (29.0)	470 (29.9)	0.02
Invasive ventilation, mean (SD), d^d^	5.9 (15.2)	3.5 (4.8)	0.21
Noninvasive respiratory support, mean (SD), d^e^	13.8 (16.5)	16.7 (16.6)	0.18
Bronchopulmonary dysplasia^f^	205 (6.6)	97 (6.2)	0.02
Patent ductus arteriosus^g^	105 (3.4)	54 (3.4)	0
Spontaneous or focal intestinal perforation	14 (0.4)	5 (0.3)	0.02

Abbreviation: SMD, standardized mean difference.

^a^ An SMD value of 0.10 was regarded as the reference value for assessing group differences.

^b^ Transient tachypnea (>60 breaths per minute) for at least 3 hours without fulfilling the criteria of respiratory distress syndrome.

^c^ Defined as hypoxemia, tachypnea (>60 breaths per minute), and characteristic radiographic findings.

^d^ Based on 598 and 204 infants needing any invasive ventilation in the no probiotics and probiotics groups, respectively.

^e^ Based on 2765 and 1482 infants needing nasal continuous positive airway pressure and/or a high-flow nasal cannula in the no probiotics and probiotics groups, respectively.

^f^ Defined as a preterm infant born at less than 32 weeks’ gestation with clinically significant lung disease who required, beyond a transient period, supplemental oxygen and/or other respiratory support at 36 weeks’ postmenstrual age.

^g^ Defined as symptomatic patent ductus arteriosus treated pharmacologically or surgically with the aim of active closure.

### Outcome Rates

**Figure 1.**
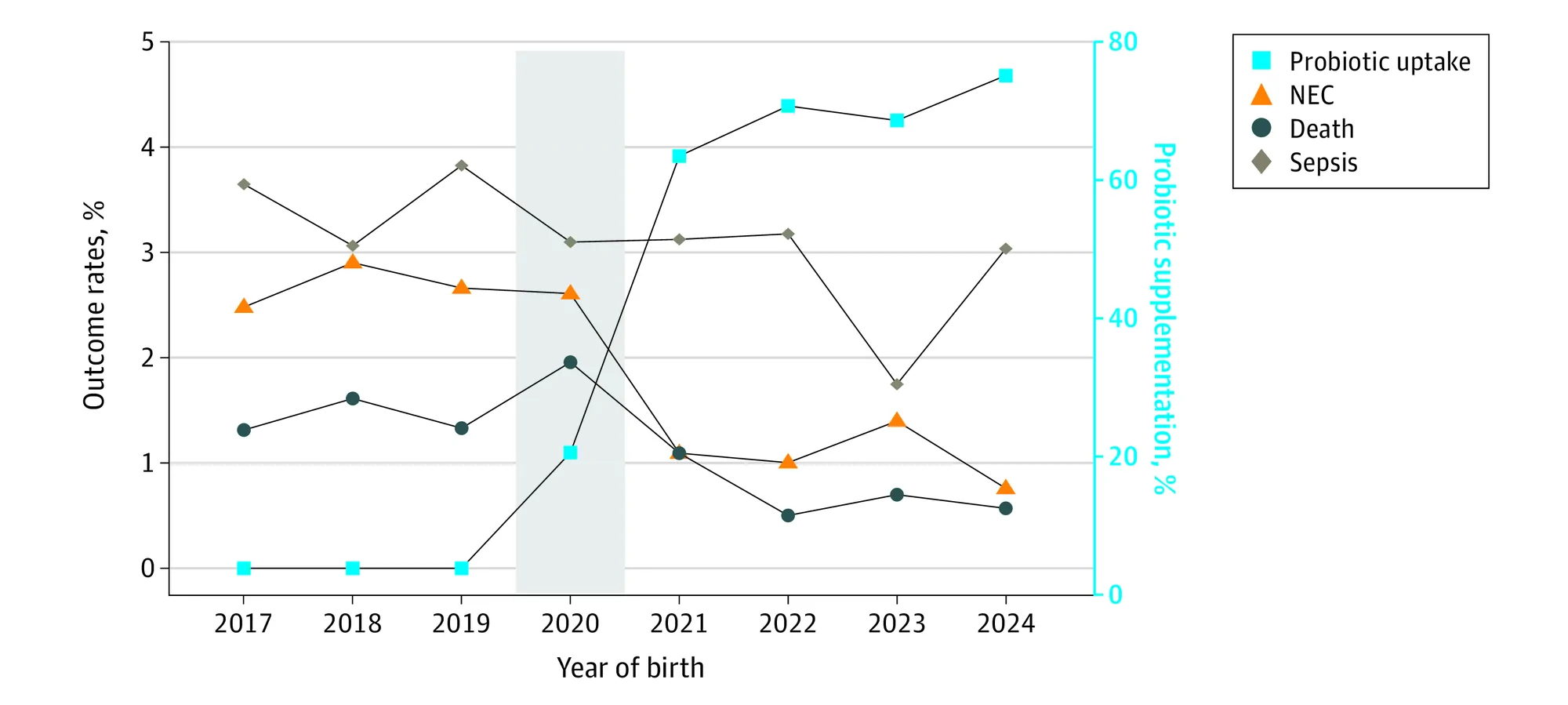
Line Graph of Temporal Trends in Probiotic Exposure and Adverse Outcomes Among Very Preterm Infants Born in Sweden in 2017 to 2024 (N = 4695) The shaded area indicates the year of introduction of the national probiotic guideline (2020). Trends are descriptive and intended to provide temporal context; adjusted associations were evaluated using regression-based and bayesian analyses. NEC indicates necrotizing enterocolitis.

Temporal trends in the rates of probiotic exposure, death, NEC, and culture-proven late-onset sepsis are shown in Figure 1. Following the national probiotics guideline in 2020, uptake increased rapidly from 0% before 2020 to 16.9% in 2020 and to a mean (SD) of 64.8% (4.7%) in 2021 to 2024. Comparing 2017 to 2019 with 2021 to 2024, ie, the periods before and after the national guideline, the overall rate of the composite outcome of death and/or NEC declined from 3.4% to 1.5%. Stratifying by probiotic exposure, the rates of the composite outcome in infants not exposed were 3.4% in 2017 to 2019 and 2.9% in 2021 to 2024, while the rate in 2021 to 2024 in infants who were exposed was only 0.8%. The rate of the composite outcome of death and/or late-onset sepsis was 4.5% in 2017 to 2019 and 3.5% in 2021 to 2024.

### Outcome Risks

Infants supplemented with probiotics had lower relative risks of the composite outcome of death and/or NEC (adjusted relative risk [ARR], 0.28 [95% CI, 0.14-0.56]) compared with those not supplemented (Table 2). When analyzed separately, probiotic exposure was associated with lower risk of both NEC (ARR, 0.25 [95% CI, 0.10-0.61]) and death (ARR, 0.16 [95% CI, 0.06-0.45]). Similar risk reductions were observed in stratified analyses, including infants born at gestational age 28 to 29 weeks (NEC: ARR, 0.25 [95% CI, 0.10-0.64]; death: ARR, 0.20 [95% CI, 0.05-0.76]) and 30 to 31 weeks (NEC: ARR, 0.35 [95% CI, 0.15-0.81]; death: ARR, 0.09 [95% CI, 0.01-0.68]) (eTable 3 in Supplement 1). In analyses restricted to surgical NEC (Bell stage 3), probiotic exposure was associated with a particularly large risk reduction (ARR, 0.04 [95% CI, 0.01-0.21]) (Table 2).

**Table 2.** Probiotic Supplementation and Rates and RRs of Death and/or Sepsis and Death and/or NEC in Very Preterm Infants, Sweden 2017-2024 (N = 4695)

Outcome	Infants, No. (%)		RR (95% CI)	
	No probiotics (n = 3124)	Probiotics (n = 1571)	Crude^a^	Adjusted^b^
**Death and/or NEC**				
Composite	106 (3.4)	13 (0.8)	0.24 (0.14-0.44)	0.28 (0.14-0.56)
Subcomponent				
Death	45 (1.4)	4 (0.3)	0.18 (0.07-0.43)	0.16 (0.06-0.45)
NEC (Bell stage 2-3)	78 (2.5)	10 (0.6)	0.29 (0.15-0.57)	0.25 (0.10-0.61)
**Death and/or surgical NEC**				
Composite	73 (2.3)	5 (0.3)	0.14 (0.06-0.32)	0.12 (0.05-0.30)
Subcomponent				
Death	45 (1.4)	4 (0.3)	0.18 (0.07-0.43)	0.16 (0.06-0.45)
Surgical NEC (Bell stage 3)	40 (1.3)	1 (0.1)	0.05 (0.01-0.29)	0.04 (0.01-0.21)
**Death and/or sepsis**				
Composite	137 (4.4)	54 (3.4)	0.78 (0.58-1.06)	0.86 (0.58-1.26)
Subcomponents				
Death	45 (1.4)	4 (0.3)	0.18 (0.07-0.43)	0.16 (0.06-0.45)
Sepsis	96 (3.1)	50 (3.2)	1.04 (0.73-1.47)	1.22 (0.78-1.90)

Abbreviations: NEC, necrotizing enterocolitis; RR, relative risk.

^a^ Controlled for clustering by hospital.

^b^ Adjusted for gestational age (days), birth weight (Fenton *z* score), Apgar score at 5 minutes (numerical), surfactant treatment (yes or no), and birth year (numerical) and controlled for clustering by hospital.

**Figure 2.**
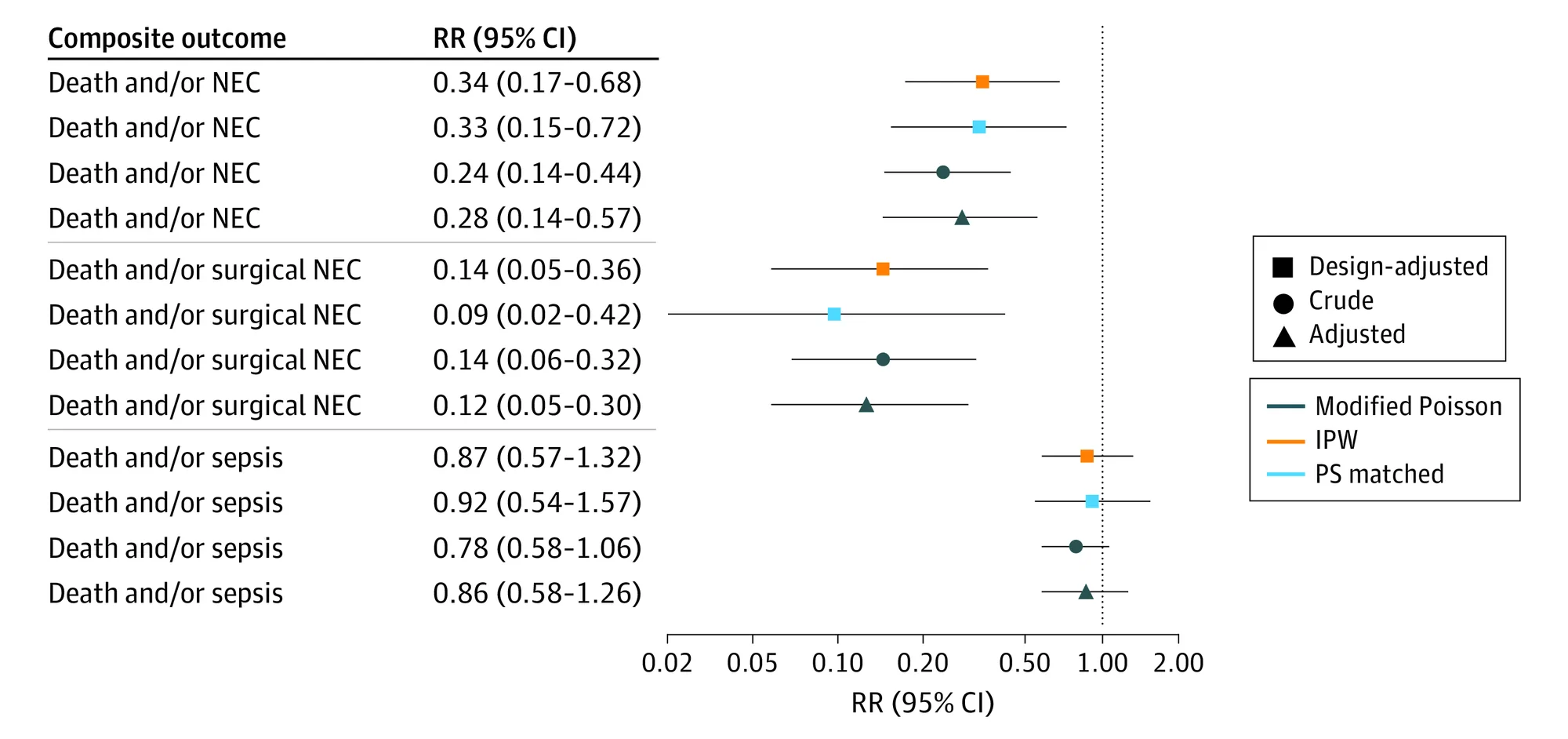
Dot Plot of the Relative Risks (RRs) of Adverse Composite Outcomes Associated With Probiotic Exposure Across Analytic Approaches Poisson regression models were adjusted for gestational age, birth weight *z* score, 5-minute Apgar score, surfactant treatment, and year of birth, with clustering by hospital. The vertical dashed line indicates RR = 1.00. IPW indicates inverse probability weighting; NEC, necrotizing enterocolitis; PS, propensity score.

Probiotic exposure was not associated with risk of death and/or culture-proven late-onset sepsis (rate, 3.4% vs 4.4%; ARR, 0.86 [95% CI, 0.58-1.26]) (Table 2). Regarding probiotics-associated sepsis, SNQ did not collect data on sepsis caused by probiotic bacteria. However, the overall risk of culture-proven sepsis remained similar in groups not exposed and exposed to probiotics (ARR, 1.22 [95% CI, 0.78-1.90]). Furthermore, we surveyed all Swedish NICUs during this study, and all reported no cases of probiotic-associated sepsis. Replication analyses using a PS-matched cohort and inverse probability weighting yielded similar results for all 3 composite outcomes (Figure 2; eTable 4 in Supplement 1).

**Figure 3.**
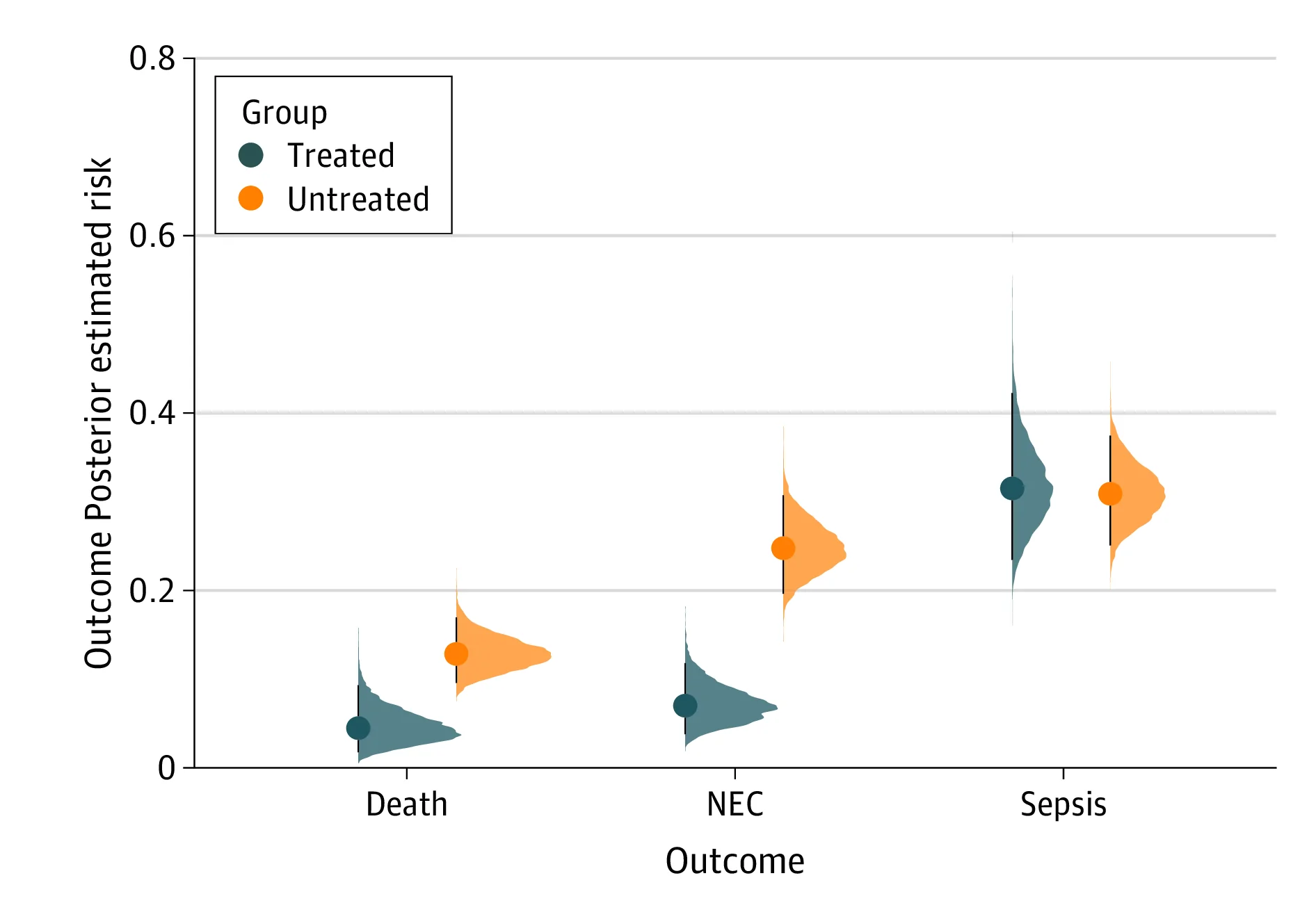
Posterior Marginal Risk Estimates Plot for Death, Necrotizing Enterocolitis (NEC), and Sepsis From Joint Multivariate Bayesian Modeling Estimated absolute risks are shown as posterior means with 95% credible intervals (shading) under model-based scenarios in which all infants were assumed to receive probiotics (treated) or not to receive probiotics (untreated). The estimates are derived from a bayesian model that simultaneously analyzed all 3 outcomes while adjusting for gestational age, birth weight *z* score, Apgar score at 5 minutes, surfactant treatment, hospital, and birth year. Modeling the outcomes jointly allowed the analysis to account for correlations between related neonatal complications.

Next, we fitted a joint multivariate bayesian model to estimate the effects of probiotic exposure on NEC, mortality, and sepsis while accounting for correlations among those outcomes. Posterior estimations from the joint bayesian model showed lower risks of NEC and mortality under probiotic exposure, with no corresponding change in estimated sepsis risk (Figure 3).

To explore how probiotics exposure influenced mortality, we extended the joint bayesian model to include NEC as a mediator (estimator) of death, allowing probiotic effects on mortality to operate through NEC-mediated and NEC-independent (nonmediated) pathways. Probiotic exposure showed a clear association with reduced mortality (posterior mean risk difference, −0.008 [95% credible interval, −0.013 to −0.003]). Exploratory mediation analyses suggested that a substantial proportion of the estimated mortality reduction associated with probiotic supplementation was mediated through a lower NEC incidence (42.3% [95% credible interval, 16.1%-100%]) (eFigure 2 and eTable 5 in Supplement 1).

## Discussion

This cohort study found that implementation of a multistrain probiotic as the standard of care for very preterm infants in Swedish NICUs was associated with a reduced risk of death and/or NEC, while the risk of culture-proven late-onset sepsis remained unchanged. Following introduction of the national probiotic supplementation guideline, there was a rapid rise in the high rate of use, comparable to rates in Canada and Europe. In the Canadian Neonatal Network, the rate of uptake among infants born at less than 34 weeks’ gestation was reported to be 67% in 2022.^9^ In England and Wales, use among infants born at less than 32 weeks’ gestation was 54% in 2022.^15^ In the US, probiotic use has historically been lower. For example, the Vermont Oxford Network reported that exposure was 13% in 2019.^29^ Furthermore, the policy context shifted in 2023 after the US Food and Drug Administration warned against probiotic products without regulatory approval.^30^ Following these actions, most US NICUs halted probiotic use.^31^

Overall, our findings are consistent with the broader literature linking the use of probiotics to a lower risk of mortality and NEC in preterm infants. The magnitude of the observed risk reduction was larger than reported in previous studies^9,15^ and may reflect high implementation fidelity or contextual factors in Swedish neonatal care. While a Cochrane review did not provide definitive evidence, several subsequent network and umbrella meta-analyses reported that probiotics are generally associated with lower risks of NEC and mortality, even though this association is strain dependent.^4,10,32^ In a large Canadian study,^9^ probiotic use (mostly a bifidobacterial multistrain product of *Bifidobacterium breve*, *Bifidobacterium bifidum*, *Bifidobacterium longum *subspecies* infantis*, and *B longum *subspecies* longum*) was associated with lower risks of all-cause mortality and the composite outcome of NEC and/or mortality among infants born at less than 34 weeks’ gestation. In England and Wales, probiotic use (mostly a multistrain product of *Lactobacillus acidophilus*, *B bifidum*, and *Bifidobacterium infantis* or the same multistrain product used in Sweden) was associated with lower risk of severe NEC (confirmed at laparotomy or postmortem or listed as primary cause of death), including in infants younger than 28 weeks’ gestation.^33^ Interestingly, a quality improvement initiative at a US level 4 NICU that used the same strains as implemented in Sweden found lower NEC rates, and discontinuation of probiotics following the aforementioned regulatory actions^30^ was followed by a subsequent rebound in NEC rates.^31^ Finally, a recently published placebo-controlled randomized clinical trial based on a single-strain product (*Lactobacillus reuteri*) reported that all-cause mortality was reduced (6.2% vs 8.5%, respectively) while NEC rates were similar (8.7% vs 10.2%, respectively).^34^

While probiotic supplementation was associated with a lower risk of NEC, we found no corresponding association for culture-proven late-onset sepsis. That finding aligns with the ProPrems randomized clinical trial in Australia and New Zealand using the same strains,^8^ while, for example, the aforementioned cohort study in England and Wales reported that probiotics were associated with a sepsis risk reduction of 6%.^33^ One may speculate about those inconsistencies, whether they reflect variations in disease definitions, study populations, or probiotic strains used. Regarding probiotic-associated sepsis, no cases during the implementation period were reported when we surveyed Swedish NICUs, which is reassuring and supports the safety of this strategy. Nonetheless, potential risks remain,^30,35^ such as bacteremia from translocation in high-risk infants and contamination during handling of products. Such risks highlight the importance of continued safety surveillance, local microbiology services having appropriate methods to detect administered strains, and clear guidance on targeted antimicrobial therapy when probiotic organisms are suspected.^13^ Although ongoing vigilance remains warranted, our findings support a favorable benefit-risk profile for probiotic supplementation in very preterm infants, which could have a large population health benefit. Families should be informed about this positive benefit-risk profile.^13^

### Strengths and Limitations

Our study’s main strength is its population-based design and large sample size based on a national registry of standardized and prospective data with high validity. These study characteristics allowed us to estimate clinically relevant risks before and after the implementation of probiotic supplementation outside the controlled conditions of a randomized clinical trial. The baseline NEC rate in our study was relatively low compared with published benchmarks,^1^ which we attributed to established process improvement efforts, including nutritional practices within Sweden’s long-standing breastfeeding-supportive environment.^36^ Despite this favorable baseline, probiotic administration was still associated with a large reduction in NEC. The observed association was consistent in direction and magnitude across different analytic methods, supporting robustness in a clinical setting.

We also acknowledge several limitations. While regional guidelines related to enteral nutrition did not substantially change during the study period, the retrospective, observational, and unblinded design may have introduced information bias and residual confounding. The incidence of NEC is known to fluctuate across periods. However, the incidence was similar throughout the study period in infants not receiving probiotic supplementation. In addition, our findings remained consistent after controlling for birth year, reducing the likelihood that observed associations were solely due to temporal trends. The small differences between crude RRs and ARRs support the robustness of the observed associations between probiotic exposure and reduced risk of death and/or NEC. Another limitation was that misclassification of NEC diagnoses could not be excluded in our study population.^37,38^ In a validation study by Challis et al,^23^ medical record review showed a 28% reduction in registry-recorded NEC cases, suggesting that our NEC incidence could be overestimated by a similar magnitude. However, in our composite outcomes, NEC was defined as either any NEC (Bell stage 2-3) or surgical NEC (Bell stage 3), and analyses including only surgical NEC cases, with an assumingly more stringent NEC diagnosis, yielded similar results. Implementation of a new guideline may lead to indication bias if probiotic supplementation would be systematically targeted to very preterm infants perceived to be either at lower or higher risk, distorting the observed association between probiotics and outcomes.^39^ We cannot exclude the possibility of selection bias. A substantial proportion of infants were not supplemented with probiotics since the national recommendation in 2020, but the baseline characteristics of infants given and not given probiotics were similar. Finally, we had no data on ethnicity and social characteristics and, therefore, could not evaluate potential social determinants of health.

## Conclusions

This cohort study found that implementation of a multistrain probiotic to very preterm infants was associated with a reduced risk of death and/or NEC. These population-based findings may help inform shared decision-making between clinicians and families. Importantly, our results should not be generalized to extremely preterm infants (<28 weeks’ gestation), a population with much higher baseline risks of adverse outcomes.

## Notes

Supplement 1. **eAppendix. **Expanded Bayesian Analysis and Mortality Decomposition **eTable 1. **Mortality and Recorded Causes of Death **eFigure 1. **Directed Acyclic Graph (DAG) of the Association Between Probiotics Supplementation and the Composite Outcomes of Death, Necrotizing Enterocolitis (NEC) and/or Sepsis **eTable 2. **Characteristics of Very Preterm Infants Born in Sweden in 2017-2024, Born in 2017-2019 Before the National Probiotic Guideline, and Born in 2020-2024, Not Supplemented or Supplemented With Probiotics **eTable 3. **Probiotics Supplementation and Rates and Relative Risks of Death and/or Sepsis and Death and/or Necrotizing Enterocolitis (NEC) in Very Preterm Infants Born in Sweden in 2017-2024, Stratified by Gestational Age **eTable 4. **Relative Risk Estimates From Alternative Analytical Approaches **eFigure 2. **Model-Based Decomposition of the Probiotic-Associated Mortality Reduction **eTable 5. **Model-Based Decomposition of the Probiotic-Associated Mortality Reduction

Supplement 2.
Data Sharing Statement
