# Supplementary material for: Probiotics and Risk of Death, Necrotizing Enterocolitis, and Sepsis in Very Preterm Infants
Source: JAMA Netw Open. 2026 Sep 23;9(9):e2635560. doi: 10.1001/jamanetworkopen.2026.35560 (PMC13602013; doi:10.1001/jamanetworkopen.2026.35560)
Supplement: Supplementary file 1 [file jamanetwopen-e2635560-s001.pdf]

Supplemental Online Content

Mitha A, Söderquist Kruth S, Sinervo E, et al. Probiotics and risk of death, necrotizing enterocolitis, and sepsis in very preterm infants. *JAMA Netw Open*. 2026;9(9):e2635560. doi:10.1001/jamanetworkopen.2026.35560

eAppendix. Expanded Bayesian Analysis and Mortality Decomposition

eTable 1. Mortality and Recorded Causes of Death

eFigure 1. Directed Acyclic Graph (DAG) of the Association Between Probiotics Supplementation and the Composite Outcomes of Death, Necrotizing Enterocolitis (NEC) and/or Sepsis

eTable 2. Characteristics of Very Preterm Infants Born in Sweden in 2017-2024, Born in 2017-2019 Before the National Probiotic Guideline, and Born in 2020-2024, Not Supplemented or Supplemented With Probiotics

eTable 3. Probiotics Supplementation and Rates and Relative Risks of Death and/or Sepsis and Death and/or Necrotizing Enterocolitis (NEC) in Very Preterm Infants Born in Sweden in 2017~~20~~-2024, Stratified by Gestational Age

eTable 4. Relative Risk Estimates From Alternative Analytical Approaches

eFigure 2. Model-Based Decomposition of the Probiotic-Associated Mortality Reduction

eTable 5. Model-Based Decomposition of the Probiotic-Associated Mortality Reduction

This supplemental material has been provided by the authors to give readers additional information about their work.

© 2026 Mitha A et al. *JAMA Network Open*.

eAppendix. Expanded Bayesian Analysis and Mortality Decomposition

To complement the primary regression analyses, we fitted a joint Bayesian model for NEC, mortality, and sepsis. The model simultaneously estimated outcome risks while adjusting for gestational age, birth weight Z-score, Apgar score at 5 minutes, surfactant treatment, hospital, and birth year. Posterior risk estimates~~predictions~~ were generated under counterfactual scenarios in which all infants were assigned probiotic supplementation ("Treated") or no probiotic supplementation ("Untreated"), while keeping all other covariates unchanged. Differences between these estimated~~predicted~~ risks were interpreted as adjusted risk differences associated with probiotic exposure.

To further explore the relationship between probiotics, NEC, and mortality, we extended the model by including NEC as an estimator ~~predictor~~ of mortality. This allowed the estimated mortality reduction associated with probiotics to be decomposed into a NEC-mediated component and a residual direct component not operating through NEC. Posterior distributions of the total effect, direct effect, NEC-mediated effect, and proportion mediated are shown in Supplement eFigure 2 and eTable 5.

The model estimated an overall mortality risk reduction of approximately 0.8 percentage points associated with probiotic supplementation (posterior median risk difference −0.008, 95% credible interval −0.013 to −0.003). The NEC-mediated component accounted for a substantial proportion of this reduction (posterior median proportion mediated 42.3%, 95% credible interval 16.1%–100%), although uncertainty around the mediated fraction remained considerable. These analyses should be regarded as exploratory and are intended to illustrate potential pathways through which probiotic supplementation may influence mortality risk.

© 2026 Mitha A et al. *JAMA Network Open*.

eTable 1. Mortality and Recorded Causes of Death (n=49) in the study population of 4,695 live-born very preterm infants (28 – 31 gestational weeks), live-born at Swedish hospitals in 2017 – 2024, excluding deaths during the first three days of life or deaths due to congenital or chromosomal abnormalities.

**Cause of death**	**n (%)**
Asfyxia	4 (8.2)[1]
Intracranial bleeding	4 (8.2)[2]
Respiratory	5 (10.2)[3]
Necrotizing enterocolitis (NEC)	18 (36.7)[4]
Infection	3 (6.1)[5]
Prematurity	15 (30.6)[6]
Other	14 (28.6)[7]

[1]Of these 4 infants, 4 infants had 6 other recorded causes of death (respiratory (1), necrotizing enterocolitis (1), prematurity (3), other factor (1))

[2]Of these 4 infants, 2 infants had 3 other recorded causes of death (prematurity (2), other factor (1))

[3]Of these 5 infants, 5 infants had 8 other recorded causes of death (asphyxia (1), infection (1), prematurity (4), other factor (3))

[4]Of these 18 infants, 7 infants had 8 other recorded causes of death (asfyxia (1), infection (2), prematurity (5))

[5]Of these 3 infants, 3 infants had 3 other recorded causes of death (respiratory (1), necrotizing enterocolitis (2))

[6]Of these 15 infants, 14 infants had 18 other recorded causes of death (asfyxia (3), intracranial bleeding (2), respiratory (3), necrotizing enterocolitis (5), other (5))

[7]Of these 14 infants, 6 infants had 10 other recorded causes of death (asphyxia (1), respiratory (3), intracranial bleeding (1), prematurity (5))

© 2026 Mitha A et al. *JAMA Network Open*.

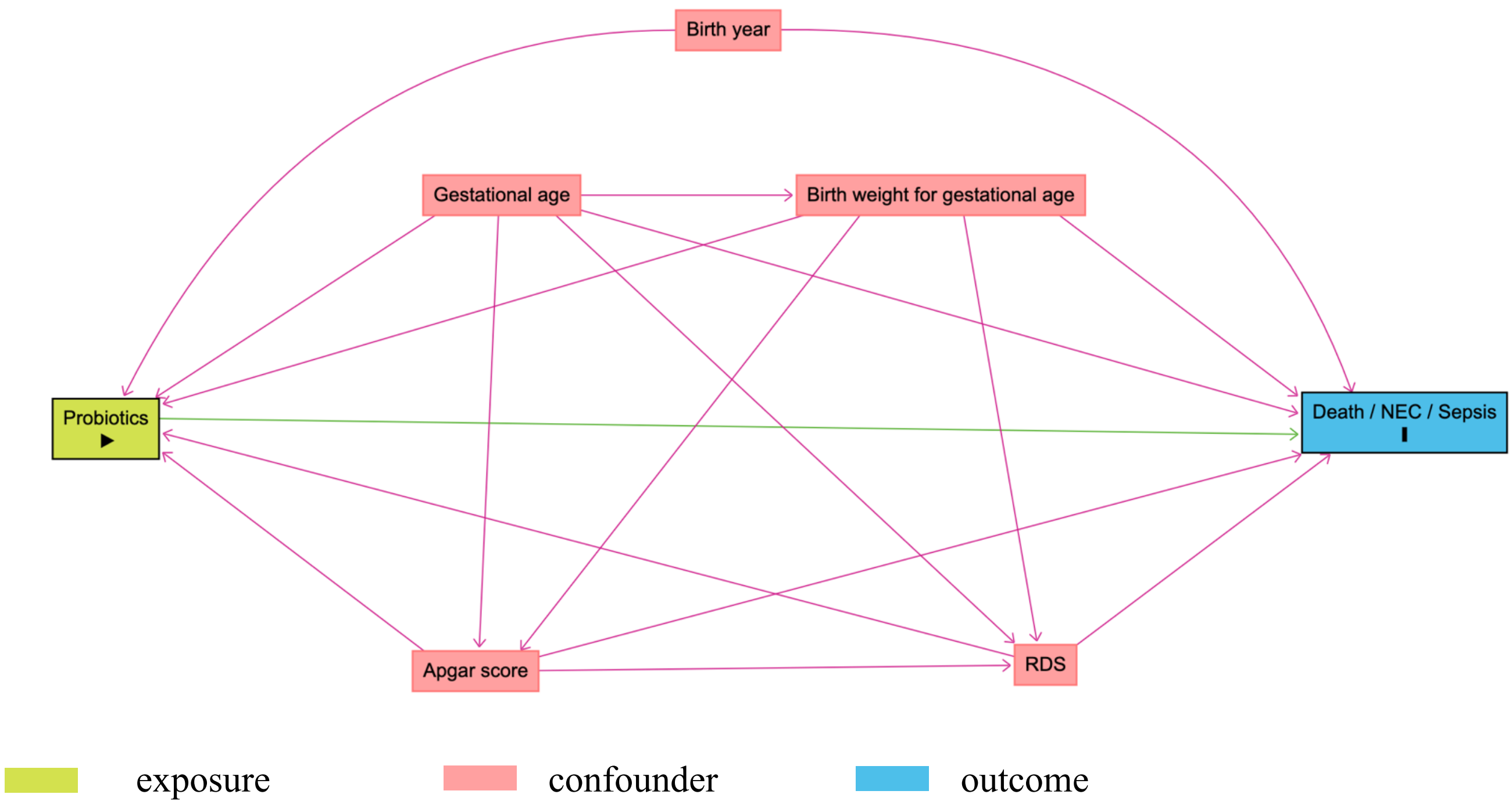

eFigure 1. Directed Acyclic Graph (DAG) of the Association Between Probiotics Supplementation and the Composite Outcomes of Death, Necrotizing Enterocolitis (NEC) and/or Sepsis

© 2026 Mitha A et al. *JAMA Network Open*.

eTable 2. Characteristics of Very Preterm Infants Born in Sweden in 2017-2024, Born in 2017-2019 Before the National Probiotic Guideline, and Born in 2020-2024, Not Supplemented or Supplemented With Probiotics

	Before probiotics 2017-2019 (n=1835)	No probiotics 2020-2024 (n=1,289)	Probiotics 2020-2024 (n=1,571)
**Perinatal characteristics**[a]			
Gestational age, weeks (SD)	30.2 (1.1)	30.4 (1.1)	30.2 (1.1)
Birth weight, Fenton Z-score (SD)	-0.21 (1.47)	-0.20 (1.42)	-0.33 (1.29)
Birth weight for gestational age, n (%)			
Appropriate	1535 (83.7)	1,076 (83.5)	1,345 (85.6)
Large	76 (4.1)	42 (3.3)	31 (2.0)
Small	224 (12.2)	171 (13.3)	195 (12.4)
Infant sex			
Male, n (%)	997 (54.3)	689 (53.4)	864 (55.0)
Female, n (%)	838 (45.7)	600 (46.6)	707 (45.0)
Singleton, n (%)	1351 (73.6)	976 (75.7)	1,194 (76.0)
Vaginal delivery, n (%)	523 (28.6)	383 (29.8)	472 (30.1)
Birth hospital, n (%)			
Level-2	1063 (57.9)	790 (61.3)	860 (54.7)
Level-3	772 (42.1)	499 (38.7)	711 (45.3)
Apgar score < 7 at 5 minutes, n (%)	358 (19.5)	232 (18.0)	213 (13.6)
Apgar score < 4 at 5 minutes, n (%)	50 (2.7)	34 (2.6)	47 (3.0)
Transient tachypnea, n (%)	1072 (58.4)	787 (61.1)	878 (55.9)
Respiratory Distress Syndrome, n (%)	913 (49.8)	667 (51.8)	774 (49.3)
Surfactant treatment, n (%)	498 (27.1)	409 (31.7)	470 (29.9)
Invasive ventilation[b], days (SD)	6.3 (18.2)	5.3 (8.3)	3.5 (4.8)
Non-invasive respiratory support[c], days (SD)	13.9 (16.5)	13.6 (16.4)	16.7 (16.6)
Bronchopulmonary Dysplasia, n (%)	151 (8.2)	54 (4.2)	97 (6.2)
Patent Ductus Arteriosus, n (%)	78 (4.2)	27 (2.1)	54 (3.4)
Spontanous / Focal Intestinal Perforation, n (%)	6 (0.3)	8 (0.6)	5 (0.3)

Abbreviations: SD, standard deviation.
[a] presented as mean value and standard deviation, or number of infants and proportion
[b] based on 372, 226 and 204 infants needing any invasive ventilation in the Before probiotics, No probiotics and Probiotics groups, respectively
[c] based on 1626, 1139 and 1482 infants needing nasal Continous Positive Airway Presssure and/or High Flow Nasal Cannula in the Before probiotics, No probiotics and Probiotics groups, respectively

© 2026 Mitha A et al. *JAMA Network Open*.

eTable 3. Probiotics Supplementation and Rates and Relative Risks of Death and/or Sepsis and Death and/or Necrotizing Enterocolitis (NEC) in Very Preterm Infants Born in Sweden in 2017~~2020~~-2024, Stratified by Gestational Age

	No probiotics n (%)	Probiotics n (%)	Crude RR[a] [95% CI]	Adjusted RR[b] [95% CI]
**28-29 weeks (N=1802[c])**				
Death/NEC	59 (5.1)	7 (1.1)	0.21 [0.12 – 0.38]	0.25 [0.11 – 0.59]
- Death	24 (2.1)	3 (0.5)	0.22 [0.08 – 0.66]	0.20 [0.05 – 0.76]
- NEC (Bell stage 2-3)	44 (3.8)	5 (0.8)	0.20 [0.11 – 0.38]	0.25 [0.10 – 0.64]
**30-31 weeks (N=2893[d])**				
Death/NEC	47 (2.4)	6 (0.6)	0.27 [0.10 – 0.77]	0.30 [0.11 – 0.80]
- Death	21 (1.1)	1 (0.1)	0.10 [0.02 – 0.64]	0.09 [0.01 – 0.68]
- NEC (Bell stage 2-3)	34 (1.7)	5 (0.5)	0.31 [0.11 – 0.86]	0.35 [0.15 – 0.81]

Abbreviations: SD, standard deviation.

[a]controlled for clustering by hospital
[b]adjusted for gestational age (days), birth weight (Fenton Z-score), Apgar score at 5 min (numerical), surfactant-treatment (yes/no) and birth year (numerical), and controlled for clustering by hospital; formal interaction analyses did not provide evidence of effect modification by gestational age (interaction p = 0.74)

[c]in infants born at 28-29 weeks, 1156 had no probiotics and 646 had probiotics

[d]in infants born at 30-31 weeks, 1968 had no probiotics and 925 had probiotics

© 2026 Mitha A et al. *JAMA Network Open*.

eTable 4. Relative Risk Estimates From Alternative Analytical Approaches

Relative risk estimates (RR) and 95% confidence intervals (CI) for the composite outcomes death and/or NEC, death and/or surgical NEC, and death and/or culture-proven sepsis obtained using modified Poisson regression with cluster-robust standard errors (CR2), propensity score matching, and inverse probability weighting.

Outcome	Method	Model	RR [95% CI]
Death / NEC	Modified Poisson (CR2)	Crude	0.24 [0.14–0.44]
Death / NEC	Modified Poisson (CR2)	Adjusted	0.28 [0.14–0.56]
Death / NEC	PS matched (1:1)	Crude (matched)	0.33 [0.15–0.72]
Death / NEC	IPW (stabilized)	Adjusted (IPW)	0.34 [0.17–0.68]
Death / sepsis	Modified Poisson (CR2)	Crude	0.78 [0.58–1.06]
Death / sepsis	Modified Poisson (CR2)	Adjusted	0.86 [0.58–1.26]
Death / sepsis	PS matched (1:1)	Crude (matched)	0.92 [0.54–1.57]
Death / sepsis	IPW (stabilized)	Adjusted (IPW)	0.87 [0.57–1.32]
Death / surgical NEC	Modified Poisson (CR2)	Crude	0.14 [0.06–0.32]
Death / surgical NEC	Modified Poisson (CR2)	Adjusted	0.12 [0.05–0.30]
Death / surgical NEC	PS matched (1:1)	Crude (matched)	0.09 [0.02–0.42]
Death / surgical NEC	IPW (stabilized)	Adjusted (IPW)	0.14 [0.05–0.36]

© 2026 Mitha A et al. *JAMA Network Open*.

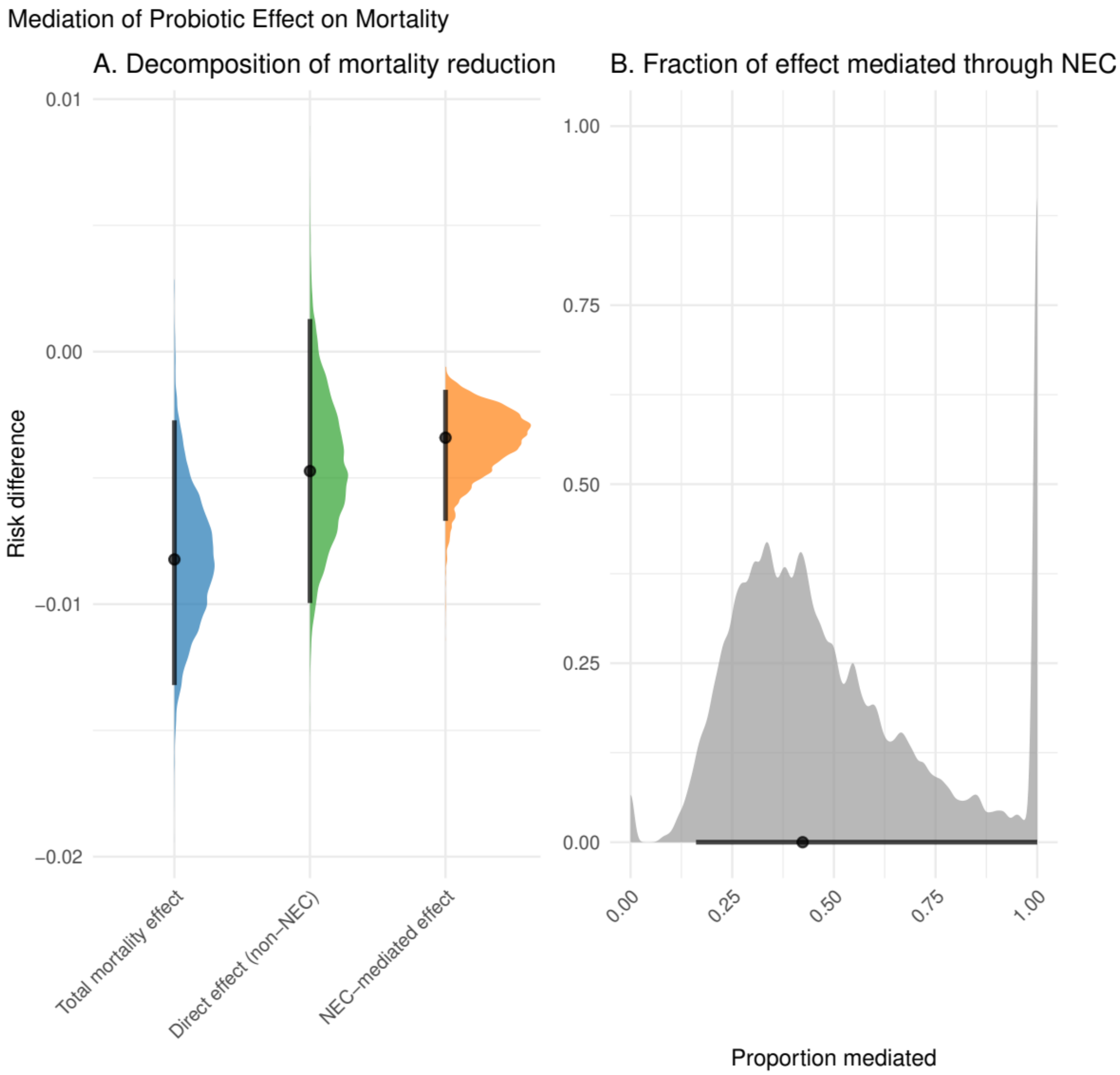

eFigure 2. Model-Based Decomposition of the Probiotic-Associated Mortality Reduction
Posterior distributions from a Bayesian decomposition analysis in which NEC was included as a mediator of the association between probiotic supplementation and mortality. (A) Posterior distributions of the total mortality effect, the direct (non-NEC-mediated) effect, and the NEC-mediated effect, expressed as absolute risk differences. (B) Posterior distribution of the proportion of the total mortality effect attributable to reduced NEC incidence. The posterior median proportion mediated was 42% (95% credible interval 16%–100%). These analyses should be interpreted as exploratory and are intended to describe potential pathways through which probiotic supplementation may influence mortality risk.

© 2026 Mitha A et al. *JAMA Network Open*.

eTable 5. Model-Based Decomposition of the Probiotic-Associated Mortality Reduction

The total estimated mortality reduction was decomposed into an NEC-mediated component and a residual direct component. Posterior median estimates and 95% credible intervals are shown. The median proportion of the mortality reduction mediated through reduced NEC incidence was 42% (95% CrI 16%–100%).

estimand	median	mean	l95	u95
Total mortality effect	-0.01	-0.01	-0.01	0
Direct effect (non-NEC)	-0.01	-0.01	-0.01	0
NEC-mediated effect	0	0	-0.01	0
Proportion mediated	0.42	0.47	0.16	1

l95 and u95 denote the lower and upper limits of the 95% credible interval, respectively.

© 2026 Mitha A et al. *JAMA Network Open*.
