# Supplementary material for: Probiotics and Risk of Death, Necrotizing Enterocolitis, and Sepsis in Very Preterm Infants
Source: JAMA Netw Open. 2026 Sep 23;9(9):e2635560. doi: 10.1001/jamanetworkopen.2026.35560 (PMC13602013; doi:10.1001/jamanetworkopen.2026.35560)
Supplement: Supplementary file 2 [file jamanetwopen-e2635560-s002.pdf]

Data Sharing Statement

Data
Data available: No
